# Recent advances in the pathology of prodromal non-motor symptoms olfactory deficit and depression in Parkinson’s disease: clues to early diagnosis and effective treatment

**DOI:** 10.1007/s12272-021-01337-3

**Published:** 2021-06-19

**Authors:** Yeojin Bang, Juhee Lim, Hyun Jin Choi

**Affiliations:** 1 College of Pharmacy and Institute of Pharmaceutical Sciences, CHA University, Pocheon, Gyeonggi-do 11160 Republic of Korea; 2 College of Pharmacy, Woosuk University, Wanju, Jeollabuk-do 55338 Republic of Korea

**Keywords:** Depression, Hyposmia, Neurogenesis, Non-motor, Parkinson’s disease

## Abstract

Parkinson’s disease (PD) is a progressive neurodegenerative disease characterized by movement dysfunction due to selective degeneration of dopaminergic neurons in the substantia nigra pars compacta. Non-motor symptoms of PD (e.g., sensory dysfunction, sleep disturbance, constipation, neuropsychiatric symptoms) precede motor symptoms, appear at all stages, and impact the quality of life, but they frequently go unrecognized and remain untreated. Even when identified, traditional dopamine replacement therapies have little effect. We discuss here the pathology of two PD-associated non-motor symptoms: olfactory dysfunction and depression. Olfactory dysfunction is one of the earliest non-motor symptoms in PD and predates the onset of motor symptoms. It is accompanied by early deposition of Lewy pathology and neurotransmitter alterations. Because of the correlation between olfactory dysfunction and an increased risk of progression to PD, olfactory testing can potentially be a specific diagnostic marker of PD in the prodromal stage. Depression is a prevalent PD-associated symptom and is often associated with reduced quality of life. Although the pathophysiology of depression in PD is unclear, studies suggest a causal relationship with abnormal neurotransmission and abnormal adult neurogenesis. Here, we summarize recent progress in the pathology of the non-motor symptoms of PD, aiming to provide better guidance for its effective management.

## Introduction

Parkinson’s disease (PD) is a progressive neurological disorder characterized by motor dysfunction that affects 10 million people globally, and this number is expected to double by 2030 (Dorsey et al. 2007). Many non-motor PD symptoms, including loss of smell, sleep disorders, depression, and constipation, can precede motor symptoms by several years. Dopamine replacement strategies are widely used for symptomatic therapy for PD as they improve key motor symptoms including bradykinesia, rigidity, and tremor. However, non-motor symptoms usually do not respond to motor deficits-targeting dopamine replacement therapy (Ray Chaudhuri et al. 2018; Deuel and Seeberger 2020). Leaving non-motor symptoms untreated can lead to a poor disease prognosis and a negative effect on the quality of life of patients with PD (Sauerbier et al. 2016). Although dopaminergic pathology is the cardinal feature in the brains of patients with PD, a more diffuse pathology might be associated with non-motor symptoms as well; the cholinergic glutamatergic, noradrenergic, and serotonergic systems (Brandão et al. 2020). Therefore, understanding the pathology of PD’s non-motor symptoms and ensuring an early and accurate diagnosis and an appropriate therapeutic approach in the PD prodromal stage remains a major and challenging goal for PD treatment. Using neuropathological PD findings as gold standard, the accuracy for a PD clinical diagnosis was only 26% in untreated or not clearly medication-responsive subjects, and 53% in early PD patients (< 5 year’s duration) responsive to medication (Adler et al. 2014).

The olfactory deficit shows high prevalence in patients with PD, early and easy diagnosis, and persistence throughout the disease course. Olfactory dysfunction has a prevalence > 90% in patients with PD and is a potential preclinical biomarker and a cardinal prodromal symptom that may precede neuropathology (Bohnen et al. 2008b; Shill et al. 2021). Although a correlation between olfactory dysfunction and neurodegenerative disorders has been increasingly recognized (Bahuleyan and Singh 2012), the underlying mechanism is not completely understood.

Depression is a nonspecific symptom, but the most common psychiatric symptom in PD, occurring in over one-third of cases. Depression may be present throughout all PD stages. For instance, at disease onset, up to 40% of patients with PD experience depression, whereas in the advanced stage, up to 70% of patients will have presented with depressive symptoms (Aarsland et al. 2009). Additional research has shown that the average onset of depressive symptom was 17.6 years earlier than the average age at PD diagnosis (Seritan et al. 2019). If PD onset could be recognized early, disease progression could be slowed by initiating appropriate neuroprotective treatment at the most effective stage. Depression could be one of the effective clinical markers of prodromal PD (Hustad and Aasly 2020), but the pathophysiology of depression in PD remains poorly understood. In the present review, we summarize the current progress in two pathological features of PD–olfactory deficits and depression–to provide crucial insights into the requirements of early diagnosis and clearer recommendations for PD treatment.

## Olfactory dysfunction

**Fig. 1:**
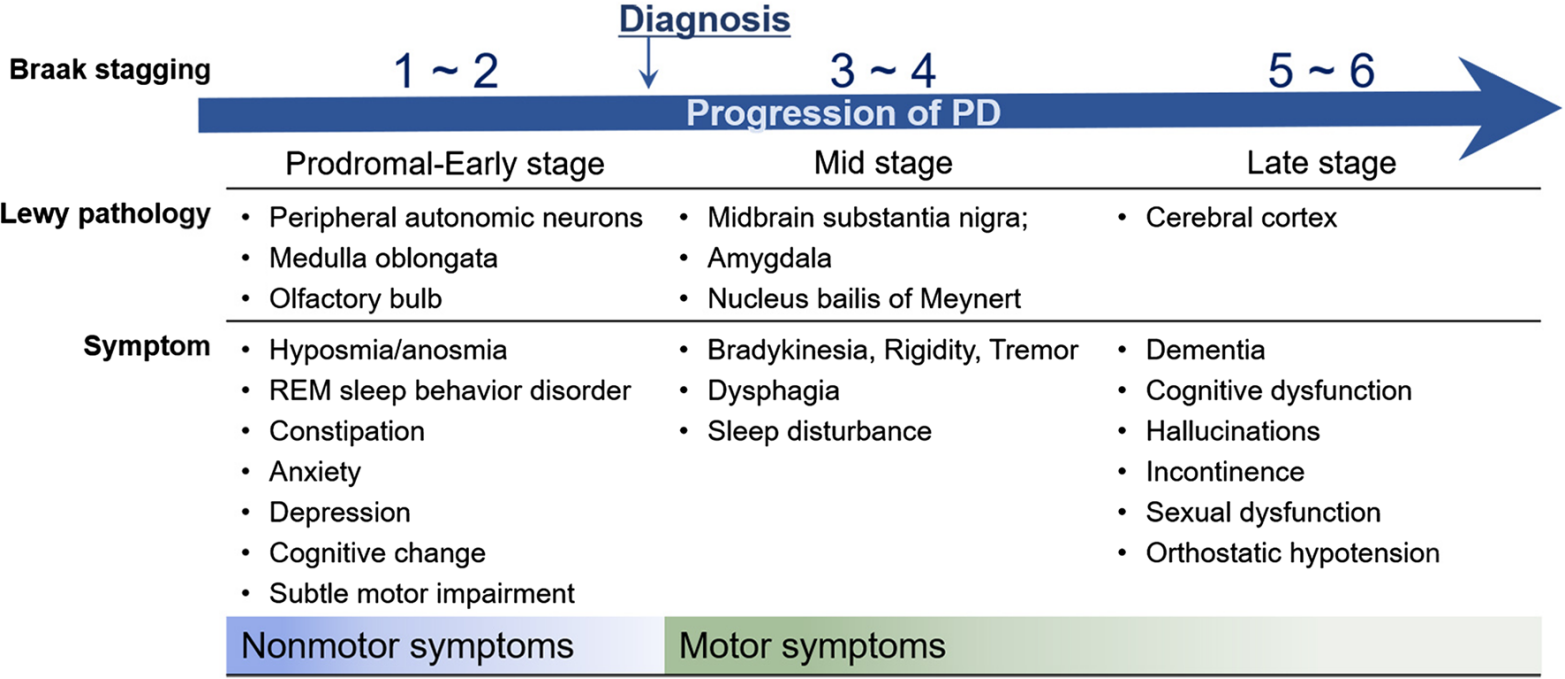
Timeline of Parkinson’s disease

Hyposmia is one of the characteristic non-motor signs of early PD, which may occur early, before the onset of motor disorders (Fig. 1). Clinical and experimental evidence suggest pathological changes in the olfactory bulb (OB), such as formation of pathological protein aggregates and changes in neurotransmitter signaling, at relatively early stages of PD, suggesting that olfaction may be vulnerable from early stages of PD progression (Table 1) (Rey et al. 2018). Olfactory dysfunction is correlated with disease progression and cognitive decline in PD (Domellöf et al. 2017; Cecchini et al. 2019). Furthermore, the effective levodopa dose is higher in patients with PD with hyposmia than in patients with normosmia (He et al. 2020). Therefore, detection of olfactory impairment could be useful for accurate PD diagnosis in the prodromal stage and for predicting disease progression risk.

**Table 1:** Pathological features of olfactory deficits with PD model

Subjects			Pathology of olfactory dysfunction		References
			Measuring olfactory function	Pathological changes	
PD patients		Control (n = 58), IPD (n = 110)		↑ LBs in OB in PD patients No significant change of OB volume (early disease stage)	Braak et al. (2003)
		Control (n = 7), IPD (n = 7)		↓ Neuron in the AON in PD patients	Pearce et al. (1995)
		Healthy age-matched control (n = 9), IPD (n = 11)	Sniffin’ Sticks test (TDI score—significant)	No significant correlation of OB volume	Mueller et al. (2005)
		Control (n = 29), IPD (n = 29)	Japanese T&T Olfactometer Test (significant)	↓ OB volume in PD patients; positive correlation with olfactory performance ↓ OS depth in PD patients; no significant correlation with olfactory performance	Wang et al. (2011)
		Control (n = 16), IPD (n = 16)	Sniffin’ Sticks test (TDI score—significant)	↓ OB volume in PD patients ↓ Left OB height in PD patients	Brodoehl et al. (2012)
		Control (n = 31), IPD (n = 52)	Sniffin’ Sticks test (TDI score—significant)	No significant correlation of OB volume	Paschen et al. (2015)
		Control (n = 19), IPD (n = 28; stage 1 or stage 2)	UPSIT (significant)	No significant correlation with UPDRS ↑ OB volume in PD patients, but ns	
		Control (n = 25), IPD (n = 59)	CC-SIT (significant)	No significant correlation between CC-SIT and disease duration	Kim et al. (2007)
		Control (n = 10), IPD (n = 10)		↑ Dopaminergic neurons in OB in PD patients	Huisman et al. (2004)
		Control (n = 16), early IPD (n = 12)	Sniffin’ Sticks test (TDI score—significant)	↓ Neuronal activity in the amygdala and hippocampus in PD patients ↑ Neuronal activity of the dopaminergic, cortico-striatal loops in PD patients	Westermann et al. (2008)
		Control (n = 25), IPD (n = 7)	Sniffin’ Sticks test (TDI score—significant)	No significant correlation of olfactory epithelium	Witt et al. (2009)
		Early PD	UPSIT (significant)	Positive correlation of DAT uptake with olfactory performance	Siderowf et al. (2005), Berendse et al. (2011) and Oh et al. (2018)
		Control (n = 27), early PD (n = 27)	UPSIT (significant)	↓ DAT uptake in striatum; positive correlation with olfactory performance in PD patients	Bohnen et al. (2007)
		Early PD without dementia (n = 58)	UPSIT (significant)	Positive correlation of acetylcholinesterase activity with olfactory performance in the hippocampal formation, amygdala and neocortex	Bohnen et al. (2010)
		Control with nonsmokers (n = 53) and smokers (n = 17), PD with nonsmokers (n = 54) and smokers (n = 22)	UPSIT (significant)	No significant correlation with olfactory performance among control ↑ Olfactory performance in smokers among PD	Lucassen et al. (2014)
PD animal models	Transgenic	Alpha-SYN^A53T^ mice (aged 2–8 months)		↑ Alpha-synuclein deposits in tertiary olfactory structures in α-SYN^A53T^ mice	Ubeda-Bañon et al. (2012)
		Alpha-SYN mice (aged 4–9 months, α-synuclein expression in the brain but no loss of nigrostriatal dopamine neurons)		No significant correlation in odor detecting and habituating ↑ Olfactory impairment in α-SYN mice ↑ Proteinase K-resistant α-synuclein inclusions in OB in α-SYN mice	Fleming et al. (2008)
		Alpha-SYN mice (aged 8–12 months)		↓ Olfactory function in α-SYN mice ↓ Glomerular dopaminergic neurons in GL α-SYN mice ↑ Alpha-synuclein in non-dopaminergic cells in GCL α-SYN mice ↓ Maximum mitochondrial respiration in OB synaptosomes α-SYN mice	Kim et al. (2011)
		Alpha-SYN^A53T^ mice (aged 6–10 months)		↓ Odor discrimination and odor detection in α-SYN^A53T^ mice ↓ Cholinergic neurons in MCL in α-SYN^A53T^ mice ↓ Acetylcholinesterase activity in GL in α-SYN^A53T^ mice ↑ Dopaminergic neurons in GL in α-SYN^A53T^ mice	Zhang et al. (2015a)
		Alpha-SYN mice (aged 4 months)		↓ Neurogenesis and neurons in OB and hippocampus α-SYN mice	Winner et al. (2004, 2008)
		PINK1^−/−^ mice (aged 27 months)		↓ Odor discrimination in PINK^−/−^ mice ↓ Serotonergic innervation in GL in PINK^−/−^ mice	Ferraris et al. (2009)
		Parkin^−/−^ mice (aged 18 months)		↓ Norepinephrine in OB in Parkin^−/−^ mice	Von Coelln et al. (2004)
		LRRK2^G2019S^ mice		↓ Newborn neuron in GL and GCL in LRRK2^G2019S^ mice ↓ Dopaminergic neurons in GL in LRRK2^G2019S^ mice	Winner et al. (2011)
	Neurotoxin	MPTP (20 mg/kg, ip, 4 times at 2 h intervals, sacrificed 1, 3 and 5 days after the last injection)		↑ Microgliosis in OB in MPTP treated mice	Vroon et al. (2007)
		MPTP (30 mg/kg, sc, 1 time, sacrificed 1.5, 4 or 8 h after injection) and nicotine (0.33—1 mg/kg, ip, 4 times prior to after MPTP)		↓ Striatal MPP + in nicotine treated mice	Quik and Monte (2001)
		MPTP (14 mg/kg, ip, 3 weeks daily, sacrificed 1.5, 4 or 8 h after injection) and nicotine (1 mg/kg, ip, daily prior to 2–8 h intervals MPTP)		↑ Odor discrimination and odor detection in nicotine treated mice ↑ Choline acetyltransferase in OB in nicotine treated mice	Yang et al. (2019)
		MPTP (0.5–8 mg/kg, iv, once every 2 weeks)		↑ DA in OB of MPTP treated monkeys No significant correlation of DA metabolites, serotonin and their metabolites, noradrenaline and amino acid neurotransmitters aspartate, glutamate, taurine and γ-aminobutyric acid in OB in MPTP treated monkeys	Pifl et al. (2017)

This table summarizes pathological characteristics changes in PD patients and animal models. For details and references, see main text

AON, Anterior olfactory nucleus; CC-SIT, crosscultural smell identification test; DA, dopamine; DAT, Dopamine transporter; GL, Glomerular layer; GCL, Granule cell layer; ip, Intraperitoneally; iv, intravenously; LBs, Lewy bodies, LRRK2, leucin-rich repeat kinase 2; MPTP, 1-methyl-4-phenyl-1,2,3,6-tetrahydropyridine; MCL, Mitral cell layer; OB, Olfactory bulb; OS, Olfactory sulcus; PD, Parkinson’s disease; sc, Subcutaneous; SYN, synuclein; UPSIT, University of Pennsylvania Smell Identification Test; UPDRS, Unified Parkinson's disease rating scale

### Olfactory ability and olfactory atrophy in PD

Patients with PD are often unaware of their olfactory deficit before testing. Less than 25% of patients with olfactory disturbance seem to realize their problem prior to diagnosis (Schmidt et al. 2020). Some patients with PD misestimate their own olfactory function as better than their actual odor identification ability (Leonhardt et al. 2019). Olfactory disturbance negatively affects the quality of life, by impacting the enjoyment of food, mood, and social interaction (Frasnelli and Hummel 2005; Vassilaki et al. 2017). Olfactory function is typically measured in a clinical setting by odor discrimination, odor identification, and odor detection threshold tasks (Fullard et al. 2017). The most frequently used and well-characterized method for olfaction assessment is odor identification by the University of Pennsylvania Smell identification Test (UPSIT), which includes 40 microencapsulated odorous substances presented in a “scratch ‘n’ sniff” booklet (Doty et al. 1984). Other olfactory performance clinical tests, such as odor detection threshold tests (e.g., Smell Threshold Test or Connecticut Chemosensory Clinical Research Center Test), can be used in combination with the UPSIT (Fullard et al. 2017). The UPSIT scores of PD were strongly correlated with various motor and non-motor symptoms, such as anxiety, depression and sleep disturbances, as well as with the degree of nigrostriatal dopaminergic cell loss, indicating that olfactory assessment using UPSIT could be a potential diagnostic tool for predicting disease progression. (Roos et al. 2019). In a few cases, olfactory deficits have also been reported in patients with familial PD (Liu et al. 2020b). Monogenic PD patients with mutations in the genes leucine-rich repeat kinase 2 (LRRK2) (Khan et al. 2005; Healy et al. 2008), PTEN-induced putative kinase 1 (PINK1) (Ferraris et al. 2009), vacuolar protein sorting 35 (VPS35) (Struhal et al. 2014), glucocerebrosidase (GBA) (Alcalay et al. 2012) showed similar changes in UPSIT scores to those with idiopathic PD, but patients with Parkin (Khan et al. 2004; Malek et al. 2016) mutations showed normal olfactory function.

The pathological relationship between olfactory deficit and decreased OB volume in PD is controversial. When analyzed using the MRI and the Japanese T&T olfactometer threshold test, the olfactory performance positively correlated with OB volumes in both patients with PD and controls (Wang et al. 2011). In patients with early stage PD, olfactory performance is positively correlated with OB volume, but not with the olfactory sulcus depth (Wang et al. 2011). Table 1 summarized other reports on the correlation between olfaction and OB volume in patients with PD. In idiopathic PD cases, the OB volume on 3-T magnetic resonance imaging (MRI) did not differ from that of healthy age-matched controls (Paschen et al. 2015). Although the UPSIT scores were significantly lower in stage 1 and 2 patients than in controls, no statistically significant difference was observed in OB volumes between PD and control groups (Hakyemez et al. 2013). Further, olfactory biopsy results of patients with PD showed no significant changes in the olfactory epithelium between patients with PD and controls (Witt et al. 2009), suggesting that olfactory deficits in PD could be due to abnormal olfactory brain transmission rather than structural damage to the olfactory system.

### Lewy pathology in the olfactory system in PD

Lewy pathology in the OB was detected in 95% of patients with PD and in 7% of elderly controls without parkinsonism diagnoses (Beach et al. 2009). In the brain of PD patients, immunoreactive Lewy bodies and Lewy neurites, which affect the olfactory system, are detected in the OB and dorsal glossopharyngeus–vagus complex even at very early stages (Gustavsson et al. 2020). These deposits spread to the brainstem including medulla and pontine tegmentum (Hawkes et al. 2010), and reach the substantia nigra in Braak stage 3, whereupon the typical motor symptoms of PD begin (Fearnley and Lees 1991). This aberrant deposit accumulation shows varying degrees of severity among neurodegenerative diseases. In postmortem tests in the olfactory region, tau-related pathology has been found in patients with Alzheimer’s disease, PD, Lewy bodies dementia, and frontotemporal dementia. However, tau-related pathology is not detected in patients with progressive supranuclear palsy or corticobasal degeneration with less olfaction loss (Doty 2017). A study on α-synucleinopathy in the olfactory system detected higher immunoreactivity against α-synuclein in the different divisions of the olfactory system in patients with PD (Braak stages 3–5) than age-matched controls. Although motor dysfunction in PD is primarily associated with the pathology in dopaminergic neurons in the nigrostriatal pathway, α-synucleinopathy along the olfactory pathway was rarely detected in dopaminergic cells, but rather in glutamate-, calcium-binding protein- and substance P-positive cells (Ubeda-Bañon et al. 2010).

### Changes in neurotransmitter signaling in PD-associated olfactory dysfunction

It has been demonstrated that alteration of neurotransmitters is associated with hyposmia in PD. Marked decreases in the numbers and activity of cholinergic neurons in the mitral cell layer and increases in dopaminergic neuron numbers and TH protein levels in the glomerular layer were reported in α-synuclein A53T transgenic mice compared to wild-type littermates (Zhang et al. 2015a). A significant increase in dopamine levels is also shown in the OB of 1-methyl-4-phenyl-1,2,3,6-tetrahydropyridine (MPTP)-induced monkey PD model (Pifl et al. 2017) without changes in other monoamine neurotransmitters. However, a decrease in norepinephrine level was detected in the OB in Parkin null mice (Von Coelln et al. 2004). In Pink1 null mice, damage to the serotonergic innervation in the olfactory glomerular layer was reported, along with an impaired fine-tuning of the smell identification function (Ferraris et al. 2009). In this section, we focus on dopamine and acetylcholine signals, the most reported neurotransmitters related to olfactory abnormalities in PD.

#### Dopaminergic signaling

**Fig. 2:**
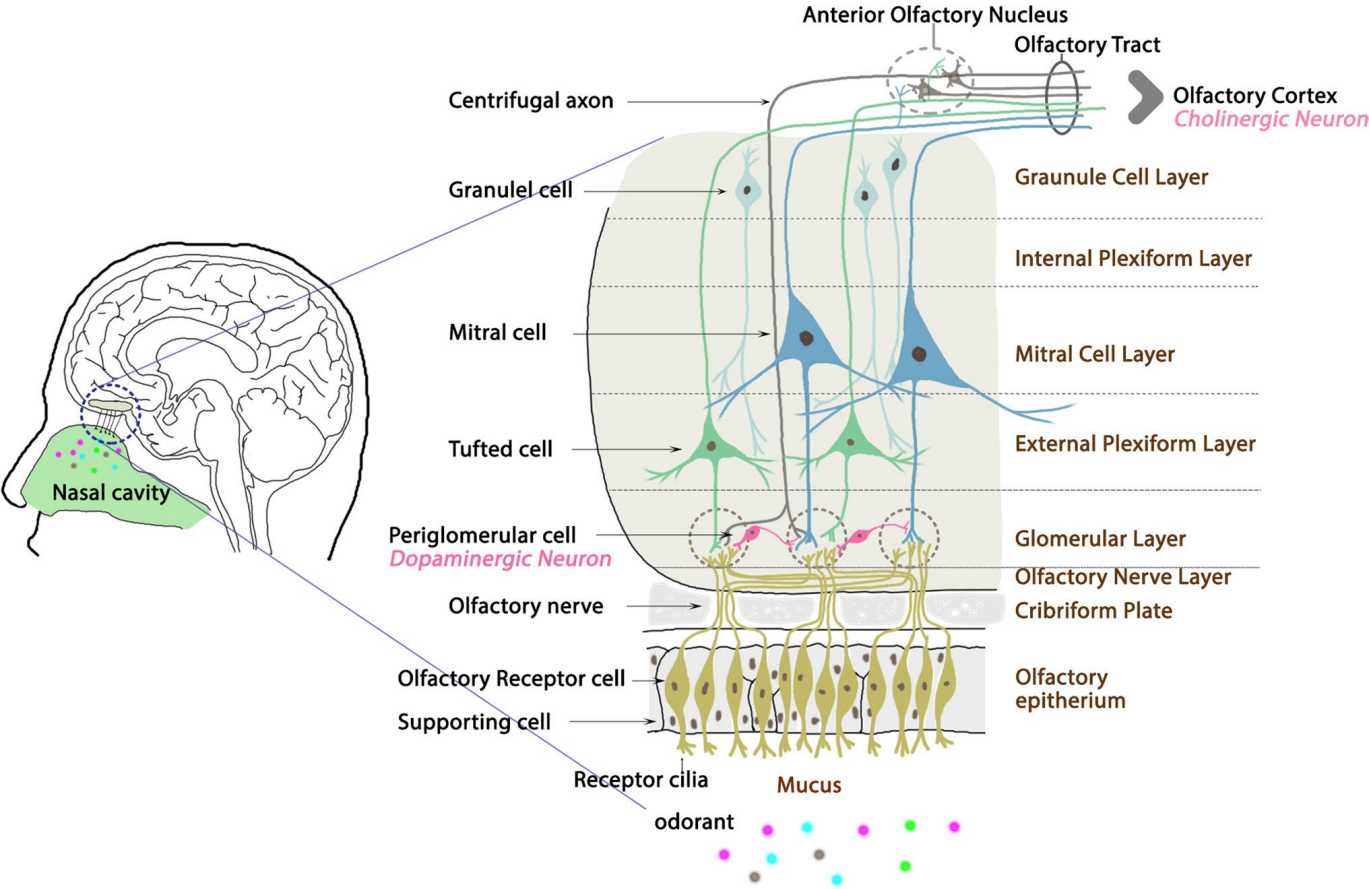
Schematic of the olfactory bulb (OB) showing the major cell types and the synaptic interaction. Note that dopamine modulates the membrane potential of the bulbar mitral cells through D2 dopamine receptors, which restrict the entry of the perceived olfactory input. The presynaptic D2 receptor functions in processing odor information and adapting the bulbar network to external stimuli

The OB glomerular layer contains up to 10% dopaminergic interneurons (Fig. 2), which participate in olfactory processes such as perception, discrimination, and olfaction-guided social interactions (Tillerson et al. 2006; Marin et al. 2017). Dopamine modulates olfactory transmission in the olfactory glomerular layer (Liu et al. 2020a) and has inhibitory regulation through D2 dopamine receptors in processing synaptic inputs from olfactory sensory neurons to mitral cells in the OB (Duchamp-Viret et al. 1997; Hsia et al. 1999; Berkowicz and Trombley 2000). In vitro studies show that activation of presynaptic D2 receptor inhibits excitatory glutamatergic transmission between mitral/tufted cells and interneurons, an effect mainly mediated by inhibition of calcium channels (Davila et al. 2003; Gutièrrez-Mecinas et al. 2005). The numbers and firing rates of dopaminergic periglomerular neurons in the OB are higher in patients with PD than age-matched controls; tyrosine hydroxylase (TH) immunoreactivity in the OB is twice as high in patients with PD than in controls (Huisman et al. 2004). Further, the olfactory impairment in PD is not responsive to dopamine replacement therapy (Huisman et al. 2004; Haehner et al. 2011). Therefore, the PD-associated dopamine alteration in the OB seems to have a pathological mechanism different from that of the PD-associated dopaminergic neurodegeneration in the nigrostriatal pathway; it may be a compensatory mechanism triggered by early degeneration of other neurotransmitter systems (Mundiñano et al. 2011).

For differential diagnosis of PD, dopamine transporter (DAT) imaging is useful. Substantial evidence shows the close correlation between abnormal DAT binding and olfactory deficits in early PD (Bohnen et al. 2007; Berendse et al. 2011). The level of DAT uptake is significantly reduced in the bilateral caudate and left anterior and posterior putamen in patients with PD with hyposmia compared to patients with normosmia (Oh et al. 2018). DAT positron emission tomography (PET) shows a difference in correlation coefficients between olfactory testing score and DAT binding potential depending on the brain region; with a higher correlation for the hippocampus than the amygdala, ventral and dorsal striatum (Bohnen et al. 2008a). These findings suggest that dopaminergic impairment in regions other than the OB could be responsible for the olfactory dysfunction observed in patients with PD.

#### Cholinergic signaling

The OB receives several neuromodulatory signals, including central cholinergic transmission. Cholinergic signals from the basal forebrain regulate neuronal activity within the OB, thus modulating olfactory function (D'souza and Vijayaraghavan 2014), further contributing to olfactory perceptual learning, odor discrimination, and odor detection (Mandairon et al. 2006; Chaudhury et al. 2009). Evidences have shown that dysregulated cholinergic neurotransmission is associated with olfactory loss in PD (Perez-Lloret and Barrantes 2016). Although the underlying mechanisms by which acetylcholine modulates neuronal excitability in OB and olfactory responses are not well understood, a substantial correlation exists between UPSIT scores and acetylcholinesterase activity in patients with PD. A cholinergic deficit occurs in the limbic archicortex of PD patients without dementia and is associated with olfactory dysfunction (Bohnen et al. 2010). This relationship is stronger than that with dopaminergic denervation in the nigrostriatal pathway. Moreover, several animal studies have demonstrated that cholinergic system disruption could cause impaired odor discrimination. Blocking nicotinic receptors in the OB of cannulated rats showed decreased spontaneous discrimination of chemically related odorants (Mandairon et al. 2006), and also cholinergic OB innervation was reduced in MPTP-monkeys compared to control animals. Further, MPTP decreased dopaminergic innervation and cholinergic neurons in the horizontal limb of the diagonal band of Broca, the nucleus where cholinergic centrifugal projections to the OB originate (Mundinano et al. 2013). OB glomeruli contain high concentrations of nicotinic acetylcholine receptors and receive strong cholinergic innervation from the basal forebrain (Ma and Luo 2012). Retrospective studies highlight nicotine’s potential to improve olfactory function in PD patients (Quik and Monte 2001; Quik et al. 2008; Nicholatos et al. 2018): among PD patients, smokers scored higher on the UPSIT than non-smokers (Lucassen et al. 2014). The ameliorative effect of nicotine on olfactory dysfunction is also shown in the MPTP-induced PD mouse model. Nicotine attenuated the deficit in odor discrimination and detection, the loss of choline acetyltransferase expression in the OB, and the loss of cholinergic neurons and dopaminergic input in the horizontal limb of the diagonal band in MPTP-treated mouse brains (Yang et al. 2019).

### Decreased olfactory neurogenesis in PD-associated hyposmia

Adult neurogenesis occurs primarily in the subventricular zone (SVZ) of the lateral ventricles and the sub granular zone of the hippocampus (Lois and Alvarez-Buylla 1993; Palmer et al. 1997). Newborn neuronal precursors generated in the adult SVZ migrate toward the OB via the rostral migratory stream (RMS), and differentiate into GABAergic and dopaminergic granule and periglomerular interneurons. In the olfactory epithelium, the number of basal stem cells decreases with age, associated with a deterioration of olfaction (Rebholz et al. 2020). Disruption of adult neurogenesis in the SVZ may contribute to diverse pathological states such as decreased neuronal plasticity, olfactory deficits, and/or cognitive dysfunction in the PD brain (Marchetti et al. 2020). BrdU-positive newborn neurons in the OB were reduced in the adult mouse brain overexpressing wild-type or mutant α-synuclein (Winner et al. 2004; Winner et al. 2008). Delayed neural stem cell migration through the SVZ/RMS/OB system and reduced neural stem cell survival is observed in the OB of human–α-synuclein transgenic mice (Winner et al. 2004; Tani et al. 2010). Mice overexpressing the PD-related mutant protein G2019S LRRK2 also display a significant decrease in the neurogenesis of dopaminergic (TH, BrdU, and NeuN positive) neurons, as well as a reduced survival of newborn neurons in the OB (Winner et al. 2011). In contrast, the number of BrdU-positive cells in the OB granule cell layer decreased in the brains of 6-hydroxydopamine-lesioned adult rats, but dopaminergic neurogenesis increased in the glomerular layer after lesioning (Winner et al. 2006).

### Clinical significance of olfactory dysfunction: biomarker for early PD

Because of its early appearance, high prevalence and easy and inexpensive assessment, olfactory dysfunction in PD could be a good early biomarker for PD. In addition, the measurement of olfactory deficits has great potential for augmenting diagnostic accuracy and distinguishing idiopathic PD from other diseases such as essential tremor, parkinsonism-associated tauopathies, atypical parkinsonian syndromes, and drug-induced parkinsonism at early stages of the disease (Ponsen et al. 2004; Baba et al. 2011; Doty 2012; Elhassanien et al. 2021).

## Depression

Depression is the most prevalent non-motor psychiatric symptom in people with PD. More than 40% of individuals with PD have symptoms of depression (Todorova et al. 2014). It has been reported that monogenic PD patients with mutations in the α-synuclein, LRRK2, VPS35, Parkin, PINK1, DJ-1 and GBA genes exhibit psychiatric disturbances such as depression and anxiety (Liu et al. 2020b). In particular, PD patients carrying parkin, PINK1 and GBA mutations have more severe depression compared with idiopathic PD (Ephraty et al. 2007; Thaler et al. 2018; Zhou et al. 2020). Currently, there are no guidelines or recommendations for selective treatment of depression associated with PD, so patients with PD are not receiving the optimal treatment for depression (Weintraub et al. 2003). Here, we would like to summarize the current knowledge of depression pathophysiology in PD (Table 2) to provide appropriate therapeutic strategies.

**Table 2:** Pathological features of depression with PD model

Subjects		Group	Pathological changes	References
PD patients		Non-depressed PD (n = 12), depressed PD (n = 8)	↓ DA in CSF in depressed PD patients	Lian et al. (2018)
		Control (n = 10), Non-depressed PD (n = 24), depressed PD (n = 10)	↑ Binding of SERT in amygdala, hypothalamus, caudal raphe nuclei, posterior cingulate cortex in depressed PD patients	Politis et al. (2010)
		Control (n = 91), Non-depressed PD (n = 43), depressed PD (n = 30)	Abnormal white matter microstructures in depressed PD patients	Li et al. (2020b)
		Non-depressed PD (n = 12), depressed PD (n = 8)	↓ Binding of DAT and NAT in limbic system in depressed PD patients	Remy et al. (2005)
PD animal models	Transgenic	Alpha-SYN^A53T^ mice (aged 6, 9 and 12 months)	Depressive behavior (↑ immobility time in TST and FST) in α-SYN^A53T^ mice ↓ 5-HT, ↓NA in hippocampus in α-SYN^A53T^ mice ↑ MAO-A in hippocampus in α-SYN^A53T^ mice	Li et al. (2020a)
		Alpha-SYN rats (aged 6 weeks)	Depressive behavior (↓ sucrose preference in SPT) in α-SYN rats ↓ BDNF and spinophilin in SN of α-SYN rats	Caudal et al. (2015)
		LRRK2^G2019S^ mice (aged 9–19, 43–52, 65–83 weeks)	Depressive behavior (↑ immobility time in TST and FST, ↓ sucrose preference in SPT) in LRRK2^G2019S^ mice ↑ 5-HT1aR in hippocampus, amygdala, dorsal raphe nuclei in LRRK2^G2019S^ mice	Lim et al. (2018b)
		Parkin^−/−^ mice (aged 5–6 months)	Depressive behavior (↑ immobility time in TST and FST) in Parkin^−/−^ mice ↓ LTP in hippocampus in Parkin^−/−^ mice	Rial et al. (2014)
		6-OHDA (6 μg, stereotaxic)	Depressive behavior (↑ immobility time in FST, ↓ sucrose preference in SPT) in 6-OHDA administrated rat ↓ DA, ↓DOPAC in striatum in 6-OHDA administrated rats ↓ 5-HT, ↓HIAA in hippocampus in 6-OHDA administrated rats	Santiago et al. (2014)
		6-OHDA (12 μg on each side, stereotaxic)	Depressive behavior (↓ sucrose preference in SPT) in 6-OHDA administrated rat ↓ DA, DOPAC, 5-HT and HIAA in striatum in 6-OHDA administrated rat	Silva et al. (2016)
	Neurotoxin	MPTP (25 mg/kg, ip, once a day for 5 days)	Depressive behavior (↑ immobility time in FST and TST) in MPTP administrated mice ↓ BrdU- and TH-positive cells in DG in MPTP administrated mice	Zhang et al. (2016)
		MPTP (30 mg/kg, ip, once a day for 5 days)	Depressive behavior (↑ immobility time in TST) in MPTP administrated mice ↓ TH-positive cells in SN in MPTP administrated mice ↑ α-SYN (Ser 129) in SN in MPTP administrated mice	Yan et al. (2020)

This table summarizes pathological characteristics changes in PD patients and animal models. For details and references, see main text

5-HT, 5-hydroxytryptamine, serotonin; 6-OHDA, 6-hydroxydopamine; BDNF, brain derived neurotrophic factor; CSF, cerebrospinal fluid; DA, dopamine; DAT, dopamine transporter; DG, dentate gyrus; DOPAC, 3,4-Dihydroxyphenylacetic acid; FST, forced swimming test; GDNF, glial cell-derived neurotrophic factor; HIAA, 5-Hydroxyindoleacetic acid; LRRK2, leucine-rich repeat kinase 2; MPTP, 1-methyl-4-phenyl-1,2,3,6-tetrahydropyridine; NA, noradrenaline; NAT, noradrenaline transporter; NGF, nerve growth factor; NT-3, neurotrophin-3; PD, Parkinson’s disease; SERT, serotonin transporter; SN, substantia nigra; SPF, sucrose preference test; TH, tyrosine hydroxylase; TST. Tail suspension test

### Neurotransmitter alterations in depression associated with PD

Changes in neurotransmitter systems appear before dopaminergic neurodegeneration and influence the development of non-motor symptoms. PET showed lower DAT availability in striatal and limbic structures is related to depression in PD (Remy et al. 2005; Rektorova et al. 2008). In addition, the severity of depression in patients with PD and dysfunctional mesocorticolimbic dopaminergic transmission are correlated (Wei et al. 2018). Further, the dopamine level in cerebrospinal fluid was significantly lower in depression with PD patients than in non-depressed patients with PD (Lian et al. 2018). In addition, in depressed patients with PD, pathological processes in the serotonergic neuronal system, such as changes in the serotonin transporter (SERT), appeared prior to lesions in dopaminergic midbrain neurons (Pagano et al. 2017). Higher SERT levels resulted in worsening depressive symptoms, and increased SERT binding in raphe nuclei and limbic structures were found in PD patients with depression compared to those without depressive symptoms (Boileau et al. 2008; Politis et al. 2010). Reduced concentrations of plasma serotonin (5-hydroxytryptamine, 5-HT) and its metabolite 5-hydroxyindoleacetic acid (5-HIAA) also correlated with the severity of depression in PD (Tong et al. 2015). In addition, depressed patients with PD presented reductions in acetylcholine receptor binding in the cortex (Meyer et al. 2009).

Depressive behavior and abnormal neurotransmission are shown in PD animal models: both in neurotoxin-based models that induce dopaminergic neurodegeneration, and genetic models associated with mutations in PD-related genes (Blesa et al. 2012). Lesions of the nigrostriatal pathway induced by 6-hydroxydopamine (6-OHDA) cause depression-like behavioral changes similar to the premotor symptoms of PD. In this model, the striatal contents of dopamine, dopamine metabolite dihydroxyphenylacetic acid (DOPAC), 5-HT, and 5-HIAA, were all decreased (Silva et al. 2016). Another neurotoxin, MPTP, also increases depression-like behavior and decreases TH expression (Zhang et al. 2016; Yan et al. 2020). Further, upregulation of monoamine oxidase A (MAO-A) and a decrease in noradrenaline and 5-HT in hippocampus were shown in the brain of a-synuclein A53T transgenic mice (Li et al. 2020a). In the LRRK2-G2019S PD mice model, anxiety/depression-like behavior was observed before the onset of motor dysfunction, accompanied by upregulation of the serotonin 5-HT_1A_ receptor (Lim et al. 2018b). The monoamine oxidase-B (MAO-B) inhibitor selegiline significantly ameliorated depressive behavior (immobility time in the forced swim test) and restored reduced striatal 5-HT, cortical norepinephrine, and plasma corticosterone in CD157 knockout mice (Kasai et al. 2017). These findings suggest that impaired monoaminergic neurotransmission contributes to depression in PD, but the current findings do not explain all pathologies in depressed patients with PD.

### Altered neuroplasticity and neurogenesis in the PD brain

In addition to theories focusing on neurotransmitters, theories of neuroplasticity and neurogenesis have been advanced to overcome the limitations of the monoamine hypothesis. These are the leading alternate hypotheses: meta-analyses of MRI studies have shown reductions in hippocampal volume in depressed patients relative to healthy subjects (Videbech and Ravnkilde 2004). To explain why hippocampal volume decreased in depressed patients, we summarize the neuroplasticity hypothesis by focusing on morphological changes such as shortened dendrites and decreased spine number and density, and the neurogenesis hypothesis by focusing on decreased hippocampal neurogenesis.

Some monoaminergic antidepressants improve synaptic plasticity at several levels, such as alteration of brain-derived neurotrophic factor (BDNF) expression and regulation of synapse formation (Björkholm et al. 2016). BDNF is a well-known growth factor that acts as an essential antidepressant. BDNF binds with high affinity to the tropomycin receptor kinase B (TrkB) receptor; BDNF-TrkB signaling can regulate neurotransmission and enhance synaptic efficacy as well as neuronal differentiation, maintenance, survival and regeneration (Cohen-Cory et al. 2010; Park and Poo 2013). A microarray study showed decreased BDNF and TrkB expression in postmortem brains of depressed patients (Guilloux et al. 2012). Moreover, BDNF serum levels were decreased in patients with PD (Jiang et al. 2019). Consistent with these findings, some studies have shown that the therapeutic effect of PD is associated with BDNF enhancement. An increase in BDNF levels was accompanied by a favorable response to dopamine D_3_ receptor agonists, significantly improving behavioral performance and attenuating dopaminergic neuronal loss in an animal model of PD (Li et al. 2010). Moreover, MAO-B inhibitors, (-) deprenyl and rasagiline, increased neurotrophic factor levels in the cerebrospinal fluid of patients with PD (Naoi and Maruyama 2009). A negative association was found between BDNF plasma levels and severity of anxiety and depression (HAM-A and HAM-D scores) (Costa et al. 2019; Yang et al. 2020).

Various evidence demonstrates that PD-related motor and non-motor symptoms are linked to white-matter abnormalities. A whole-brain diffusion tensor imaging study showed impaired frontal and limbic white matter integrity in depressed patients with PD compared to healthy controls/and non-depressed patients with PD. Depressed patients with PD also showed microstructural damage in the left hippocampal part of the cingulum (Li et al. 2020b). In addition, depressed PD patients have abnormal baseline brain activity on MRI compared with non-depressed PD, and the amplitude of low-frequency fluctuations was significantly decreased, which was positively correlated with Hamilton Depression Rating Scale scores (Wen et al. 2013).

Studies have shown that non-motor symptoms of PD are not directly associated with neurodegenerative processes in the substantia nigra pars compacta (Marxreiter et al. 2013). Hippocampal atrophy and disrupted neurogenesis have been observed in genetic animal models and in human postmortem studies of PD (Lim et al. 2018a). In addition, impaired adult neurogenesis in the dentate gyrus of the hippocampus can possibly trigger depression (Jacobs et al. 2000; Lee et al. 2013). Treatment with the antidepressant fluoxetine increases the number of BrdU positive cells in the adult rat hippocampus (Malberg et al. 2000). In fact, medications and other treatments used for depression often enhance adult neurogenesis, and clinical trials using neurogenic compounds to treat major depressive disorder are underway (ClinicalTrials.gov Identifier: NCT01520649, NCT02695472).

### Current status and limitations of treatments for depression in PD

Because the pathophysiology of depression in patients with PD is complex and differs from patients with major depression, the treatment strategy for general mood disorders may not be effective in controlling depressive symptom in patients with PD (Seppi et al. 2019). Clinically, although the majority of PD patients with depression are receiving symptomatic treatment to control their depressive symptoms, up to 50% of them remain depressed even with treatment (Weintraub et al. 2003). We have no clear guidelines or recommendations on medication for depression in patients with PD. Serotonin reuptake inhibitors and tricyclic antidepressants (TCA) are traditionally the most administered psychiatric medications in PD and somewhat effective in treating depression in PD (Liu et al. 2013). However, their efficacy in PD is still controversial, and other side effects are being raised. In patients with PD and rodent models of PD, fluoxetine has been used as an adjuvant therapy to reduce depressive symptoms and neurodegeneration (Boggio et al. 2005; Zhang et al. 2015b). However, “extrapyramidal” symptoms are associated with fluoxetine treatment; fluoxetine was found to exacerbate tremor and dopamine depletion in a rodent pharmacological model of PD (Podurgiel et al. 2015). Amitriptyline, a commonly used TCA, can interfere with the autophagy-mediated removal of protein aggregates, which could increase the risk of neurodegenerative diseases or exacerbate existing neurodegeneration (Kwon et al. 2020). A recent study showing that serotonin 5-HT_1A_ receptor upregulation is accompanied with anxiety/depression-like behavior in PD indicates that the 5-HT_1A_ receptor could be an attractive therapeutic target for PD-associated depression (Lim et al. 2018b). For a better management of depression in PD, further research is needed to evaluate the efficacy and safety of symptomatic treatments and to identify pharmacologic targets based on the specific pathogenesis of PD-associated depression.

## Conclusion

In PD, non-motor symptoms can occur years or decades before motor symptom onset and can increase caregiver burden and significantly reduce the patient’s quality of life. Non-motor symptoms in the early/preclinical stages of PD are potentially useful biomarkers for predicting the onset of motor symptoms and diagnosing PD. Moreover, these biomarkers can also identify patients at risk of developing PD or its complications and ultimately lead to neuroprotective and disease control therapy. Although the loss of nigrostriatal dopaminergic neurons is a major neurological deficit in PD, there is accumulating evidence regarding the existence of pathologies for non-motor symptoms beyond the nigrostriatal dopaminergic system. Accordingly, dopaminergic therapy does not affect the olfactory deficit often found in the early stage of PD, and commonly used antidepressants may not be effective in treating depression in PD. As such, the therapeutic management of the non-motor symptoms of PD remains challenging. This review summarizes recent advances in the understanding of the pathology and significance of olfactory dysfunction and depression in PD and therefore provides clues for identifying novel therapeutic targets for controlling PD-associated non-motor symptoms.
